# A health intervention improves cognition in older adults at risk for Alzheimer’s Disease

**DOI:** 10.1002/alz.089964

**Published:** 2025-01-03

**Authors:** Michelle Gray, Ray G Urbina, Megan Jones, Anthony Campitelli, Joshua L Gills, Sally Paulson, Jordan M. Glenn

**Affiliations:** ^1^ University of Arkansas, Fayetteville, AR USA; ^2^ NYU Grossman School of Medicine, New York, NY USA; ^3^ St. Elizabeth Healthcare, Clinical Research Institute, Edgewood, KY USA; ^4^ Neurotrack Technologies, Redwood City, CA USA

## Abstract

**Background:**

Alzheimer’s disease (AD) is the 7^th^ leading cause of death and 6^th^ most burdensome disease among US older adults. With pharmacological treatments for AD being predominantly ill‐effective, alternative, non‐pharmacological prevention and treatment strategies warrant exploration. Personal health coaching provides individualized strategies designed to improve physical, social, and emotional health leading to positive behavior changes that may improve cognitive ability. The purpose of the present investigation was to examine the effects of a health intervention on cognitive outcomes.

**Method:**

Adults (n = 184), 60+ years, completed a 24‐month health intervention. All participants had their APOE4 status determined at baseline. Each participant completed either a monthly health coaching (HC) session or received bi‐weekly health education information email (HE). RBANS and ECOG‐12 were completed at baseline and 24 months. A repeated measures ANOVA was completed to determine changes in health intervention groups and APOE4 carrier groups over time.

**Result:**

RBANS was significantly improved after HE for most tested RBANS cognitive paradigms (Language, Immediate and Delayed Memory, and Attention) among APOE4 non‐carriers. The HC intervention significantly improved Immediate Memory, and Attention among APOE4 non‐carriers. Among APOE4 carriers, HE improved Delayed Memory and the HC intervention improved Immediate Memory. ECOG‐12 values were significantly improved for both intervention groups, but only for APOE4 non‐carriers.

**Conclusion:**

Both HC and HE produced significant improvements in many cognitive paradigms among APOE4 non‐carriers, though subjective cognitive decline was only improved in APOE4 non‐carriers. More information is needed to dissect the impetus for the changes in cognition.

